# Thanatophobia (Death Anxiety) in the Elderly: The Problem of the Child’s Inability to Assess Their Own Parent’s Death Anxiety State

**DOI:** 10.3389/fmed.2017.00011

**Published:** 2017-02-27

**Authors:** Gary Sinoff

**Affiliations:** ^1^Faculty of Social Welfare and Health Sciences, Department of Gerontology, University of Haifa, Haifa, Israel

**Keywords:** thanatophobia, elderly patients, death anxiety, anxiety, elderly

## Abstract

Thanatophobia is omnipresent in our lives. Research has shown separate but connected constructs: fear of death or fear of the dying process. The influences on death anxiety are varied including religiosity, gender, psychological state, and age. It is often assumed by the children of the elderly that the fear of death is prevalent in their parents. Daily the medical staff encounters the presence of death anxiety: from family members or the staff itself. In order to understand this phenomenon, a three-tier study was conducted on non-terminal elderly inpatients in an acute geriatric care ward. The study showed that the elderly had low levels of anxiety (scoring 4/15 on Templer’s Death Anxiety Scale) but their children scored higher for themselves (6.9/15) and for their parents (8.9/15). A regression model showed that only the presence of generalized anxiety and religiosity of parent had an effect explaining 33.6% of the variance. Death anxiety of death is usually absent in the elderly but rather they fear the dying process. On the other hand, their children do fear death, which they extrapolate onto their parents. This causes conflicts since the children prevent disclosure of relevant medical information to their parents. This has to be addressed by the staff when dealing with family members, to allow open and honest communication with their patients. The staff need to explain to the family that the elderly are not afraid of death but of the suffering from the dying process.

## Introduction

Death anxiety is present in our lives and affects each and every one of us in different ways. This phobia has been described as a feeling of dread, anxiety or fear at the thought of death, or anything to do with dying (1). This anxiety related to fear of death was termed thanatophobia by Sigmund Freund in 1915 in his seminal essays titled: *Thoughts for the Time on War and Death*. Freud believed it to be related to one’s unconscious belief in one’s own immortality. Jung in 1933 wrote that “Life is like a parable, starts at birth and ends at death. In other words, death is part of the life-cycle.” So understanding the inevitability of dying is essential to our living.

Over the years, research has shown two separate but connected constructs of death anxiety: fear of death or fear of the dying process (2). Until this day, the argument remains. Is one conceptually talking about fear of death or fear of the dying process? Death anxiety has been characterized as a conscious fear of death, a fear for the body after death, a fear of lost time, a fear of suffering, a fear of the unknown, and a fear of loneliness (3–5). In fact, Farley (6) stated that it is: “A feeling of dread, apprehension or solicitude (anxiety) when one thinks of the process of dying, or ceasing to ‘be’.”

What actually affects the presence of death anxiety is still debatable. Many variables have been proposed to influence death anxiety amongst them religiosity, gender, psychological state and age. The psychological state is a known factor to cause higher death anxiety, especially in persons suffering from generalized anxiety disorder (7, 8). It has been stated that death anxiety is probably a consequence of unresolved psychological and physical distress. Kesebir (9) showed that those with higher level of humility, that is less feeling of self-importance, actually had lower levels of death anxiety. On another note, McCarthy (10) felt that death anxiety in adults was a consequence of the struggle to psychologically separate from their parents and requiring to form an independent and individual identity. From this viewpoint, adult children struggle to psychologically separate from their parents often resulting in anxiety for death of their parent.

The protective effect of religiosity is in dispute (11, 12). In the literature, there have been reports that religiosity has a positive protective effect since one is going to meet the Supreme Being and finally be given their rewards for their life on Earth (13–15). Yet others have found it to increase the fear of death for the same reason that they will be judged in heaven for their deeds while they were on Earth, a problem cross-culturally (16–19).

Gender has also been in discussion since some reports have found greater prevalence of death anxiety in males (20), and others describe greater death anxiety in females (21, 22). So it is still unclear if gender constitutes a protective or harmful effect.

Regarding age there have been multiple reports. One is aware that the aging process entails more than just changes in one’s appearance, cognitive decline, or increased generalized aches and pains; but also knowing that one is moving inevitably closer to death (23). The presence of death anxiety is reported to peak in middle age and disappear in the elderly (20, 24, 25). Russac and colleagues (26) also found that death anxiety was high in the young adult population (20-year olds) in both sexes and then declined over time but spiked once again at 50, particularly in the female population. However, they were unable to explain this phenomenon. The fact that the level of death anxiety is age related is an important finding for the multidisciplinary staff of a geriatric health team.

The staff in a geriatric care setting encounters on a daily basis the presence of death anxiety, whether from the side of the patient and/or their family member, or from the staff itself caring with persons with life-threatening diseases.

Life experiences with death may also influence attitudes about death and dying and contribute to the levels of death anxiety. It has been argued that from the staff point of view, the relating to death may be a culturally based effect. Sharif Nia and colleagues (27) recently reviewed the problem of death anxiety amongst nursing staff and provided information that culture has a major effect on how nurses adapt to death. One study showed that amongst Egyptian nursing students, the level of death anxiety was higher than Spanish nursing students but this was explained partially in the difference in age and experience (28), a similar finding was also in a study done in America (29). The presence of high levels of death anxiety often resulted, cross-culturally, in staff avoiding providing care for dying patients whether from Israel (30), Iran (31), or Japan (32).

The very existence of death anxiety often delays the ability to make unbiased and uninfluenced decisions on the side of the patient, family, or the medical staff. This becomes particularly problematic nowadays with the intimate involvement of the middle-aged children of the elderly in the decision making process for their elderly parent. Possibly the child’s belief in the existence of death anxiety on the part of their parents may impede the flow of information, sometimes against the patient’s own rights.

Studies have examined death anxiety in the young and elderly separately, but few have related to children’s ability to proxy-assess death anxiety in their own parents. A three-tier study was done by the author and colleagues to answer this complex story (33). Initially, the level of death anxiety in the young and old was investigated. Then, whether the problem is fear of death or fear of dying process in both age groups was examined, and finally was studied whether the middle-aged children of elderly patients were able to assess correctly, by proxy, the level of death anxiety in their own parents. The working hypotheses were that the level of death anxiety would be higher in the children than the elderly, the elderly would have more fear of the dying process than actual death and finally that children would not be able to proxy-assess correctly their own parent’s death anxiety level.

In the study, 44 elderly children couplets were examined, and it was found that the elderly scored lower on Templer’s Death Anxiety Scale (DAS) (mean 4.0/15) compared to children scoring for themselves (mean 6.9/15; *P*-value < 0.01). Interestingly, children scored their own parents even higher on proxy assessment (mean 8.1/15; *P*-value < 0.001 comparing to the actual score of the elderly). All of the elderly stated that they were afraid of dying a painful death, that is the dying process, and this was also picked up their children. However, the children felt that their parents were afraid of death or dying and were worrying about this all the time. Accordingly, the children assumed that their parents were afraid to see death before them or that their parent felt that their future was bleak. In fact this is opposite to what the elderly actually report. However, these beliefs correlated with the children’s own fear of death and dying (Table 1). A multiple linear regression with score on proxy-assessment DAS scare as the dependent variable and age, religiosity, presence of generalized anxiety and depression elderly, gender, education level, holocaust survivor, level of cognitive problems and functional status of parent, and age and religiosity of child were the independent variables. Only the presence of generalized anxiety and religiosity of parent was able to explain 33.6% of the variance.

**Table 1:** **Differences on Death Anxiety Scale: parent, child, and child proxy-assessing parent**.

Question	Parent (%)	Child (%)	Child proxy-assessing parent (%)
1. I am very much afraid of dying	14	48***	46***
2. The thought of death often enters my mind	39	70**	50
3. It makes me nervous when people talk about death	23	42	50*
4. I dread to think about having an operation	34	46	80***
5. I am afraid of death	11	61***	61***
6. I am afraid of getting cancer	36	55	59
7. The thought of death bothers me	11	70***	75***
8. I am often distressed by the way time flies so very rapidly	30	54	48
9. I fear dying a painful death	100	84*	98
10. The subject of life after death troubles me greatly	5	7	14
11. I am really scared of having heart attack	39	43	59
12. I often think about how short life really is	36	50	55
13. I shudder when I hear people talk about World War III	14	35*	46**
14. The sight of a dead body is horrifying to me	7	41**	52***
15. I feel that the future holds nothing for me to fear	41	57	68*

*P < 0.05.

**P < 0.01.

***P < 0.001.

As mentioned above, the presence or belief of the presence of death anxiety may affect the ability to communicate with patients about death as they age. To understand if this was gender related, an analysis was performed, and it was shown that for both sexes those who agreed to disclose medical problems to their parents compared to non-disclosures, scored lower for their parents on proxy assessment (males 4.7/15 versus 8.4/15 and females 9.6/15 versus 10.1/15). Most of the females agreed on disclosure (52.2%) even though they scored their parents higher on proxy assessment. Interestingly with regard to gender, males who believed in non-disclosure tended to mainly prevent flow of information to their fathers (83%) and females to their mothers (56%).

In an attempt to explain this model of discrepancy, a multiple linear regression with child-assessing parent DAS score as dependent variable was performed. The only variable that was significant was the presence of generalized anxiety disorder, which the children picked up as death anxiety (*t* = 2.829, *P* < 0.01). All the other independent variables such as age, gender, religiosity, educational level, presence of depression, and cognitive decline, had no effect on the model.

## Discussion

Death anxiety is a universal and fundamental phenomenon, which affects humans to various degrees. Klein in 1948 described that actually death anxiety is one of the basic feelings of humanity and is the root to all anxiety. Humans are the only species who are aware of the limitations to life and impending death. Kübhler-Ross in her book *On Death and Dying* in 1969 (34) stated that the problem of death anxiety is more a fear of death and psychological adjustment with the dying process. She emphasized five different stages a person with end-of-life illness undergoes: denial that death is eminent, anger and resentment that others will live, bargaining to cope with death, depression when recognizing the inevitability of death and finally acceptance.

Death anxiety has been identified to have six different attributes to the concept: emotion related to fear of disappearance, cognitive acceptance of death, experiential that death anxiety is not part of one’s conscious experience, development stage with identity crises affecting the degree of death anxiety, sociocultural shaping such as western societies concealing the sick and elderly accompanied by denial of death and source motivation affecting psychological status of the individual (1).

In addition there is a need to understand whether the problem of death anxiety is anxiety about death or anxiety about the process of dying. The difference is clearly shown in two different statements: “I fear death” and “I am afraid to die” (21).

One role of increasing importance to the lives of adults in the U.S. is that of caregiving for an elderly relative. Tomer and Eliason (35) postulated that death anxiety is directly influenced by death attitudes, past- and future-related regrets and is indirectly influenced by coping processes, beliefs about one’s self and the world, and by the degree to which death heighten one’s own awareness of eventual death.

The hypothesis that death anxiety is higher in the young compared to the elderly was proven by the difference on the DAS mean scores in the above mentioned study (6.9 for children versus 4.0 for parents), a finding similar to reports in the literature (20, 24, 25). The literature reports that death anxiety peaks in middle age and decreases with increasing age, a finding supported by the author’s study. The stages of death anxiety may be summarized as follows. About age 9–10, we realize that death is final; in adolescence, we have this ingrained belief of invulnerability and immortality. Changes occur in early adulthood when one becomes a parent. By middle age, one is exposed to the finality of life with death of parents, friends, and siblings. This being the period of highest death anxiety. Finally in old age, the level of death anxiety drops even in the face of death of spouses and peers. Given that older adults are temporally closer to death and probably encounter more frequent reminders of their mortality than their younger counterparts, it may be that they have come to some level of acceptance of this inevitable reality, at least at a conscious level (23). Nevertheless, a future study needs to include grandchildren for investigating death anxiety over lifespan.

The hypothesis that parents are not afraid of death but of the dying process was supported in our study. This finding was reported by others that elderly are more worried about the death process, not of death (36). The dying process is more relevant to the parent than the actual thought of death (all were worried of dying with discomfort), and here the children were able to correctly assess their parent’s fears. The final hypothesis that the children incorrectly assess death anxiety of their own parents was verified by difference in mean DAS scores (4.0 for parent versus 8.1 for child-assessing parent, *P* < 0.001).

From findings in the child’s self-assessment compared to child-assessing parent, it was seen that the child tends to extrapolate their own beliefs onto their parents. The regression model showed that the child is able to pick up some anxious traits, but transliterated by the child into existence of death anxiety in his parent. These may explain the child’s difficulty in permitting flow of information to the parent (only 43% agreeing), in contradiction to “Patient’s Rights.” In fact, the higher the child’s self-assessment DAS score, the less likely he was going to allow the staff to relay relevant medical information to his parent. This conflict between parent and child with regard to extension of life is well known (37, 38). Schafer (39) reported that the basis is often related to the desire of the child for continuing life, whereas the parent tends to give their true feelings. There was a non-significant tendency for child with higher interpersonal relation to assess correctly his parent’s death anxiety state.

No correlation was found with religiosity, gender, and education on DAS score. Many studies in the literature have reported a bidirectional effect of religion on death anxiety, some protective (11, 12, 14, 15), and others the opposite (16, 17). Florian and Kravetz (40) felt that Judaism increased death anxiety, while others reported that Christianity decreases death anxiety (40). In the study, religiosity was examined on multiple levels: self-definition, belief in a Superior Being, and religious behavior. No evidence of religiosity effect on any of the DAS scores was found. Gender played no role in the DAS score, as was also reported in the literature (20, 24).

Elderly present with less death anxiety than their children; however, the study’s uniqueness was that it proved that children were unable to assess the absence of death anxiety in their own parents and actually tended to extrapolate their own anxiety about death onto their parents. This explains why children deny the rights of their parent to receive information from the medical staff, in contradiction to the patient’s rights.

In a related vein, it may also be that as one grows older, death becomes more of an expected or normative event, and normative or typical events are less stressful and easier to cope with (41).

The other side of the story is that the clinical staff may have their own problems in relating to death accompanied by death anxiety. In a study by Peck (42), social workers with higher death anxiety were less likely to disclose information about advance directives with patients. Viswanathan (43) reported that physicians preferred to notify next of kin by telephone after a loved one’s unexpected death and was related to the physician’s own level of death anxiety. Doctors with greater death anxiety were more likely to inform next of kin that their relative was in a critical status rather than to report that their parent had died unexpectedly. The level of death anxiety of the doctor also correlated with his own personal preferences for being informed of their loved one’s unexpected death and may result in communicating problems with families about death. Health professionals with more training in palliative care and with experience over time will lower fear of death and provide more positive attitudes about caring for the elderly (44).

Communication is an essential requirement for the preservation of trust between patients and health professionals and is subject also to legal and ethical safeguards (45). From time to time, the duty to preserve confidentiality and keep the channels of communication open can present health professionals with an ethical or legal dilemma, commonly when the patient’s children request information about their parents or their treatment. It should be clearly understood that the ethical, professional, contractual, and legal positions on confidentiality are complex and may require legal guidance for health professionals dealing with the issue of death and dying.

It is rarely parents, but the parent’s children, spouse, friends, or caregivers who contribute to difficulties in communication by preventing open channels of interaction between health providers and the elderly. In some situations, an adult child might direct their anger and frustration at the treating doctor himself. However, it is important to emphasize that on the other hand, the common law generally requires consent by the parents for disclosure of information to their own children.

In conclusion, in the face of modern medicine in twenty-first century, death still usually occurs in hospitals surrounded by medical technology, but it is still a taboo subject with euphemisms used to describe the problem. The awareness that death anxiety occurs mainly in the middle-aged children and not in the elderly themselves may help health professionals to explain to the children of the elderly the true state of their parents’ level of death anxiety, thus allowing the staff open communication channels with their elderly.

## Author Contributions

The author confirms being the sole contributor of this work and approved it for publication.

## Conflict of Interest Statement

The author declares that the research was conducted in the absence of any commercial or financial relationships that could be construed as a potential conflict of interest.
